# RFS+: A Clinically Adaptable and Computationally Efficient Strategy for Enhanced Brain Tumor Segmentation

**DOI:** 10.3390/cancers15235620

**Published:** 2023-11-28

**Authors:** Abdulkerim Duman, Oktay Karakuş, Xianfang Sun, Solly Thomas, James Powell, Emiliano Spezi

**Affiliations:** 1 School of Engineering, Cardiff University, Cardiff CF24 3AA, UK; espezi@cardiff.ac.uk; 2 School of Computer Science and Informatics, Cardiff University, Cardiff CF24 4AG, UK; karakuso@cardiff.ac.uk (O.K.); sunx2@cardiff.ac.uk (X.S.); 3 Department of Oncology, Velindre NHS Trust, Cardiff CF14 2TL, UK; solly.thomas@nhs.net (S.T.); james.powell2@wales.nhs.uk (J.P.)

**Keywords:** magnetic resonance imaging (MRI), brain tumor segmentation, U-net, region-focused selection (RFS), clinical applications, generalizability of deep learning model

## Abstract

**Simple Summary:**

In our study, we addressed the challenge of the brain tumor segmentation task using a range of MRI modalities. While leading models show proficiency on standardized datasets, their versatility across different clinical environments remains uncertain. We introduced ‘Region-Focused Selection Plus (RFS+)’, enhancing the segmentation performance for clinically defined labels like gross tumor volume in our local dataset. RFS+ integrates segmentation approaches and normalization techniques, leveraging the strengths of each approach and minimizing their drawbacks by selecting the top three models. RFS+ demonstrated efficient brain tumor segmentation, using 67% less memory and requiring 92% less training time than the state-of-the-art model. The strategy achieved better performance compared to the leading model, with a 79.22% dice score. These findings highlight the potential of RFS+ in amplifying the adaptability of deep learning models for brain tumor segmentation in clinical applications. However, further research is needed to validate the broader clinical efficacy of RFS+.

**Abstract:**

Automated brain tumor segmentation has significant importance, especially for disease diagnosis and treatment planning. The study utilizes a range of MRI modalities, namely T1-weighted (T1), T1-contrast-enhanced (T1ce), T2-weighted (T2), and fluid-attenuated inversion recovery (FLAIR), with each providing unique and vital information for accurate tumor localization. While state-of-the-art models perform well on standardized datasets like the BraTS dataset, their suitability in diverse clinical settings (matrix size, slice thickness, manufacturer-related differences such as repetition time, and echo time) remains a subject of debate. This research aims to address this gap by introducing a novel ‘Region-Focused Selection Plus (RFS+)’ strategy designed to efficiently improve the generalization and quantification capabilities of deep learning (DL) models for automatic brain tumor segmentation. RFS+ advocates a targeted approach, focusing on one region at a time. It presents a holistic strategy that maximizes the benefits of various segmentation methods by customizing input masks, activation functions, loss functions, and normalization techniques. Upon identifying the top three models for each specific region in the training dataset, RFS+ employs a weighted ensemble learning technique to mitigate the limitations inherent in each segmentation approach. In this study, we explore three distinct approaches, namely, multi-class, multi-label, and binary class for brain tumor segmentation, coupled with various normalization techniques applied to individual sub-regions. The combination of different approaches with diverse normalization techniques is also investigated. A comparative analysis is conducted among three U-net model variants, including the state-of-the-art models that emerged victorious in the BraTS 2020 and 2021 challenges. These models are evaluated using the dice similarity coefficient (DSC) score on the 2021 BraTS validation dataset. The 2D U-net model yielded DSC scores of 77.45%, 82.14%, and 90.82% for enhancing tumor (ET), tumor core (TC), and the whole tumor (WT), respectively. Furthermore, on our local dataset, the 2D U-net model augmented with the RFS+ strategy demonstrates superior performance compared to the state-of-the-art model, achieving the highest DSC score of 79.22% for gross tumor volume (GTV). The model utilizing RFS+ requires 10% less training dataset, 67% less memory and completes training in 92% less time compared to the state-of-the-art model. These results confirm the effectiveness of the RFS+ strategy for enhancing the generalizability of DL models in brain tumor segmentation.

## 1. Introduction

The global age-standardized rate (ASR) of incidence of brain cancer is 3.9 per 100,000 in males and 3.0 per 100,000 in females, while the ASR of mortality is 3.2 per 100,000 in males and 2.4 per 100,000 in females [1]. Within this context, glioblastoma multiforme (GBM) is the most prevalent malignant tumor in the brain, which is rapidly lethal with a median survival time of approximately 15 months [2]. GBM occupies 57% of all gliomas and 48% of all primary malignant types of central nervous system tumors [3]. GBM is in the group of Grade 4 in the World Health Organization (WHO) report on brain tumors [4].

Patients suffering from GBM need an accurate diagnosis and prognosis for treatment decisions. Although positron emission tomography (PET) and computed tomography (CT) are occasionally used for non-invasive diagnosis of GBM, MRI (magnetic resonance imaging) has gained widespread practice in brain imaging due to its non-ionizing, high-resolution scans, and ability to generate images with high soft tissue contrast. MRI stands out as the pivotal medical imaging method, utilizing strong magnetic fields and radiofrequency waves to create detailed images of the internal structures of the brain [5]. Different MRI modalities, including T1-weighted, T1-contrast-enhanced, T2-weighted, and FLAIR, are utilized, each highlighting various tumor characteristics such as size, location, and the presence of oedema or necrosis. MRI is utilized both prior to and following treatment for the purpose of detecting and evaluating the progression of the tumor. Additionally, segmentation and quantitative evaluation of brain tumors provide information that may lead to a better understanding of disease progression and treatment strategy [6]. 

In most cases, an expert neuroradiologist is required to carry out the precise manual segmentation of tumors. This, in contrast to manual and semi-automatic segmentation, is time-consuming, labor-intensive, and sensitive to observer bias. Conversely, automatic segmentation appears as an alternative with the capability to eliminate intra- and inter-observer segmentation variability [7]. In addition, unlike manual and semi-automated segmentation, automatic segmentation is capable of overcoming reproducibility issues. 

Although machine learning (ML) was used with hand-designed features for the brain tumor segmentation task at the beginning of the brain tumor segmentation challenge (BraTS) [8], deep learning (DL) demonstrated a better performance thanks to its capability to explore highly complex features [9]. The intensity range of MRI varies for several reasons such as differences in scanner models, manufacturers, and scan acquisition techniques. This makes the generalization of ML–DL-based segmentation approaches challenging and standardization is required in terms of the intensity of MRI. When using intensity normalization techniques, an improvement can be shown in the metrics of CNN (convolutional neural network)-based brain tumor segmentation [10]. Typically, the aim of the segmentation task on brain tumors is to delineate active tumor tissue (enhancing tumor (ET)), necrotic tissue, and edema (swelling near the tumor). Additionally, in radiotherapy, the gross tumor volume (GTV) is delineated for planning purposes, which is “the gross palpable or visible/demonstrable extent and location of the malignant growth” [11]. Duman et al. demonstrated the remarkable similarity observed between GTV and tumor core (TC) [12]. Given the challenge of distinguishing between tumor and healthy tissues due to their overlapping imaging features, multiple imaging modalities of MRI such as T1, T1-contrasted (T1ce), T2, and fluid attenuation inversion recovery (FLAIR) are typically used. The response assessment in neuro-oncology (RANO) working group provides guidelines for specific MRI modalities for GBM [13]. In clinical applications, the diversity of MRI modalities in brain tumor segmentation presents obstacles, such as the differing sequences, varying image quality, resolution, and slice thickness across different modalities, and the complexity of integrating these varied data sources for accurate tumor delineation.

Even though early applications of computer vision (CV) techniques have demonstrated success under certain conditions for similar tasks, the task of medical image segmentation remains challenging due to the difficulties of feature representation [14]. Despite this, DL approaches provide promising results in the image segmentation tasks by utilizing CNN which is a widely used technique in image processing due to its ability to learn complex patterns and features in images. For efficient tumor segmentation with CNN, additional feature extraction methods are applied [15]. Recently, transformers [16] have a wide application on CV including image segmentation tasks. Transformers, often employed either independently or in integration with CNNs, are utilized to effectively capture both local and global information in medical image segmentation, with most studies integrating the transformer architecture with the U-net or its related variations [17]. In light of countless advancements and the breadth of methodologies in computer vision, it becomes essential to classify the predominant models that are crucial for navigating the complexities of medical image segmentation tasks. A substantial number of contemporary models can predominantly be classified into two principal categories: (1) multi-class segmentation; and (2) cascaded versions of binary class segmentation, which are all-in-one, end-to-end solutions for each sub-tumor (ET, TC, and whole tumor (WT)) of brain tissue.

Multi-class segmentation is an effective method to delineate multiple classes of tumors. However, in the context of the segmentation task, binary class segmentation may present exclusive benefits compared to multi-class segmentation such as simpler optimization methodology [18]. For binary class segmentation, the multi-class segmentation problem is subdivided into three separate models, each dedicated to a specific sub-region for each class. Then, all sub-regions are segmented utilizing cascaded or basic binary class models [18,19]. Additionally, the 2D models utilizing the binary classification approach might outperform the 3D models utilizing the multi-class segmentation method [20].

There are two representations of tumor classes: labels (non-overlapping masks) and sub-regions (overlapping masks). While the labels are classified as enhancing tumor, necrotic, and edema, the sub-regions being segmented are (i) enhancing tumor (ET), (ii) tumor core (TC; enhancing tumor and necrosis), and (iii) whole tumor (WT; enhancing tumor, necrosis, and edema) [21]. Despite this, multi-class segmentation still focuses mostly on label segmentation instead of region-based techniques. However, previous studies show that optimization based on sub-regions instead of labels achieves better results [22,23,24,25]. 

Lastly, it is required to note that the generalizability of DL models can be seen as one of the prominent issues, especially in medical imaging research. For example, a state-of-the-art model was trained on the BraTS dataset but was validated on a local dataset [26]. The outcomes revealed a noticeable discrepancy, with the segmentation results of the local dataset falling short of the accomplishments seen with the BraTS dataset. Another study elucidated the significant impact of differences in MRI scanners on medical image analysis, examining datasets obtained from two distinct scanners, each encompassing 50 patients, and illustrated the potential enhancement in results achieved through the implementation of varied methodologies [27]. This established that the underlying cause of these scanner differences can be attributed to variations in MRI acquisition settings, which encompass factors such as slice thickness, matrix size, echo time, and repetition time, among others. Given these identified discrepancies and challenges in generalizability, there is an evident and pressing need for more robust research endeavors aimed at amplifying segmentation accuracy on local datasets, thereby fostering enhanced universal applicability and reliability of the models in diverse clinical settings.

This study aims to improve the versatility of DL models for brain tumor segmentation in different clinical scenarios while also prioritizing time and memory efficiency to address real-world practical limitations. The aforementioned issues guided our research to propose a novel strategy called RFS+, which represents a pioneering approach distinct from the region-focused selection (RFS) [12]. RFS covers both tumor regions and labels as input masks with only Z-score normalization for only TC/GTV. This strategy includes multi-class, multi-label, and binary class segmentation approaches rather than choosing one over another. The utilization of RFS+ allows to train a U-net [28] model on the BraTS training dataset to handle the generalizability of DL models by acquiring better segmentation results over the results of a state-of-the-art model [29] on our local dataset.

RFS+ proposes three types of approaches for image segmentation, i.e., binary class, multi-class, and multi-label. It utilizes combinations of models with different normalization techniques [30] and picks the best three models on the training dataset for each region (ET, TC, and WT) to be used with ensemble learning. Most of the research on brain tumor segmentation uses the intensity normalization of Z-Score as a pre-processing step [31]. In this paper, we employed several intensity normalization techniques as well as compared the segmentation accuracy in terms of the dice similarity coefficient (DSC) score. The contributions of this study are as follows.

1. This paper, for the first time in the literature, provides a thorough investigation of different normalization techniques of MRI scans on segmentation tasks for DL models;
2. A novel strategy called RFS+ is introduced as a versatile solution for any DL model, optimizing brain tumor segmentation through a fusion of various segmentation approaches, and normalization techniques with ensemble learning, thereby enhancing accuracy and generalizability across different datasets;
3. For each region, RFS+ method provides the best DSC score by investigating the effect of normalization techniques on U-net models. It helps in the selection of the best method for each region when the aim is to use only one model. For example, transferring the trained models with one approach (such as multi-class) on ET, TC, and WT to segment GTVs cannot always give the best results. In contrast, RFS+ gives the best model for specific contours when transferring information from one contour style (TC) to another (GTV) [12];
4. RFS+ offers ensemble learning by using models with the top three DSC scores on the training dataset from the proposed models. The segmentation outcomes, when utilizing a 2D U-net model through ensemble learning, outperform those of the state-of-the-art model, indicating a substantial enhancement in segmentation accuracy provided by RFS+. Additionally, the introduction of RFS+ led to a DSC enhancement up to 1% when compared to its predecessor;
5. A state-of-the-art model, with its original Docker image that triumphed in the BraTS 2021 challenge, was evaluated using a local dataset, addressing a notable gap as most models, trained on the BraTS training dataset, have predominantly been tested on the BraTS validation and test datasets, leaving the exploration on local datasets largely untouched. This study seeks to illuminate the generalizability of DL models by showcasing the segmentation results of a state-of-the-art DL model on local datasets, serving as a pivotal guide for future research and applications in this domain.

## 2. Related Work

The deployment of DL in medical imaging is increasingly prevalent. In the BraTS challenge, most of the applications are based on DL where the best-performing model is based on CNNs (convolutional neural networks) [8]. Encoder and decoder paths have a key role in the segmentation of brain tumors which resulted in first place in the BraTS 2018 with an asymmetric encoder–decoder architecture [23]. Winners of the following three years performed their models based on U-net architecture which utilizes the encoder–decoder path. In 2019, a two-stage cascaded U-net model was used which took the first rank on the BraTS leaderboard [24] whilst in the following year, 2020, the winner used only a 3D U-net without any major modification (called nnU-net which is a framework having self-configuration). In 2021, the winning model took first place with the same nnU-net but a larger U-net architecture, even though it used different methods to achieve the best results [29].

Magadza et al. categorized CNN architectures into four subgroups which are single pathway, dual pathway, cascaded architectures, and U-net [31]. An example of the single pathway which is a simple network with small kernels in the layers using a single path is performed by Pereira et al. [10]. On the other hand, a dual pathway is a method using two different paths to collect information from the global context (the location in the brain) and local information (visual details) in the same architecture [32]. Although cascaded architecture has several distinct types, the most noticeable type is the input cascade. The output of one CNN is given as an input to another CNN [33]. The reason for this approach is to provide another image channel for the second CNN. Another approach for cascaded architecture is hierarchical segmentation which breaks the multi-class segmentation problem into the multi-stage binary segmentation problem. The importance of this architecture is utilizing the presence of tumor sub-regions and this application provides the mitigation of the class imbalance problem. Initially, WT undergoes segmentation, followed by providing the bounding box of the segmentation to the subsequent stage. Later, the second stage performs the segmentation of TC, with the last stage of the architecture dedicated to segmenting ET. The results show that the training and inference time increased. However, a successful application of binary segmentation on cascaded networks is performed by Wang et al. [34]. Additionally, a model using a binary segmentation approach which provides an advantage of memory efficiency achieves remarkable results [20]. According to the two winners of 2020 [25] and 2021 [29] BraTS challenges, models based on U-net [28] have a successful architecture that yields promising results. These models used a multi-label approach (overlapping classes instead of individual classes). There are three different approaches for using sub-tumors, namely, multi-class (non-overlapping masks), multi-label (overlapping masks), and binary classes (individual classes). The different masks for each approach can be seen in Section 3.3.1.

Other feature extraction-based methods or transformer-based DL models are not widely utilized in clinical applications [26,35] or do not produce the top performance on the last three-year BraTS challenge [25,29,36]. Although many U-net variants are available, our research provides extensive analysis of the impact of ensemble learning for baseline variants. In terms of architectural distinctions, DeepMedic (v0.8.4) [6] employs a multi-scale 3D convolutional neural network (CNN) framework, whereas the RFS+ strategy adopts a divergent structural methodology, a factor that could significantly influence their respective performances in brain tumor segmentation. Concerning the trade-off between complexity and efficiency, the cascade U-net [24] employs a cascading mechanism to enhance segmentation accuracy, whereas RFS+ prioritizes resource efficiency, an aspect that may be pivotal for its practical implementation in clinical settings. Additionally, the RFS+ approach is crafted to exhibit adaptability across various clinical scenarios, a characteristic that appears less emphasized in the cascade U-net. In the realm of learning efficiency, the RFS+ model potentially enhances this aspect through its refined optimization techniques, in contrast to the deep supervision mechanism employed by 3D deeply supervised networks (3D-DSN) [37]. Furthermore, the capability of RFS+ to adjust effectively to diverse MRI modalities and clinical contexts may offer it a distinct advantage over 3D-DSN, especially in heterogeneous healthcare settings. 

In this research, we utilized 2D, 2.5D [38], 3D U-net, and nnU-net [25] models. The DL model inclusion criteria was based on the winners’ model (nnU-net which is based on U-net) of the last three-year BraTS challenge [25,29,36] with validated superior clinical results of U-net based models (including nnU-net) over DeepMedic (v0.8.4) [35]. The RFS [12] only makes use of the models with multi-class, multi-label, and binary class segmentation approaches for only TC/GTV. The proposed RFS+ strategy expands the RFS with the combination of different normalization techniques for each region. 

## 3. Method

This section explains the details of the proposed architectures, pre-processing steps, and hyperparameters used in the models. Additionally, further information is given about two datasets that are utilized in this paper: the BraTS 2021 dataset (training and validation) and our local dataset (STORM_GLIO).

### 3.1. The Proposed Strategy: (RFS+)

**Figure 1:**
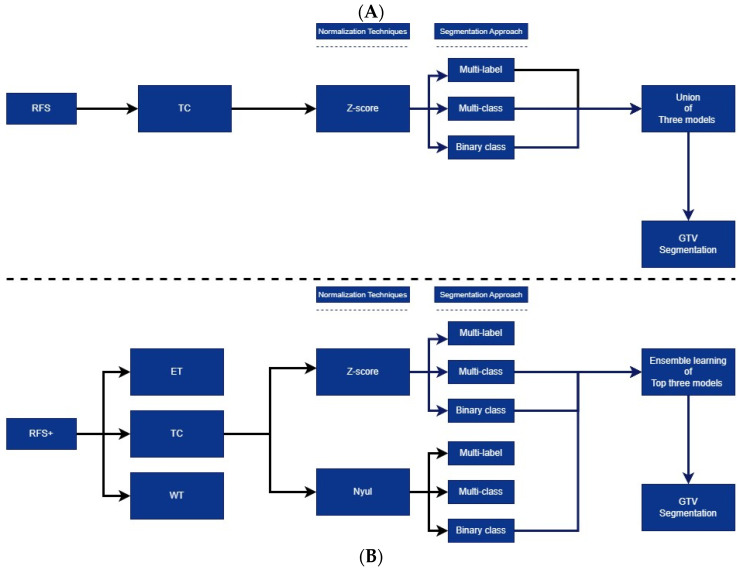
(**A**) RFS strategy and (**B**) RFS+ strategy on GTV segmentation.

The methodology depicted in Figure 1A exemplifies region-focused selection (RFS). This workflow utilizes Z-score normalization exclusively, in conjunction with three specific segmentation approaches customized for TC/GTV. The segmentation outputs from these three models are unified, encompassing overlapping areas into a unified entity. On the other hand, Figure 1B showcases ‘Region-Focused Selection Plus’ (RFS+), a versatile workflow designed for use with any DL model for brain tumor segmentation. RFS+ can target any region, be it ET, TC, or WT. For each region, additional workflows are in Supplementary Materials (see Figures S1–S3 in the Supplementary Materials).

**Figure 2:**
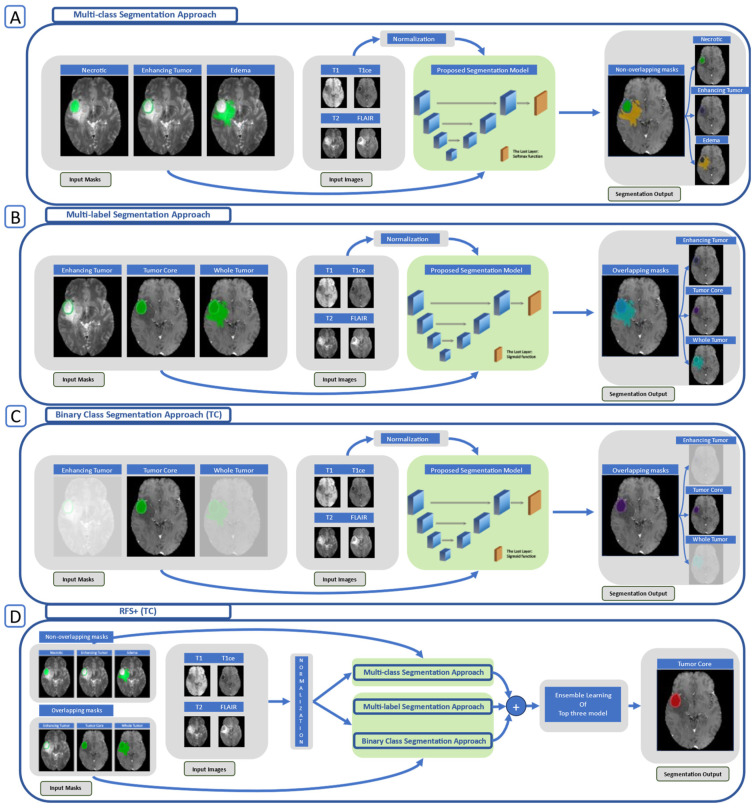
(**A**) Multi-class segmentation, (**B**) multi-label segmentation, (**C**) binary class segmentation, and (**D**) RFS+ for TC/GTV segmentation.

The distinction from RFS lies in the utilization of ensemble learning, incorporating various normalization techniques that can be customized for specific regions. This strategy consists of two main components. Firstly, various normalization techniques are applied during the pre-processing of MRI scans. Secondly, three distinct segmentation approaches are employed. When using the combinations of segmentation approaches and normalization techniques (a normalization technique such as Z-score + a segmentation approach such as multi-class), the segmentation target should be selected. We showed the transferability of a trained model on the TC contour to the GTV contour [12]. The top three models for TC/GTV on the training dataset (15% unseen dataset), which are Z-score/multi-class, Z-score/binary class, and Nyul/binary class according to DSC scores, are given to ensemble learning. A comprehensive depiction of each segmentation approach, along with their respective inputs and RFS+ strategy, is presented in Figure 2. The figures primarily illustrate the TC/GTV, but comprehensive visualizations for each segmentation, in larger dimensions, can be found in Supplementary Material (see Figures S4–S6 in the Supplementary Materials).

### 3.2. Normalization of MRI Scans

To mitigate the intensity differences between different MRI scanners, we utilized two types of normalization techniques. These are: Z-score and piecewise linear histogram matching (Nyul) which were selected from a larger set as described in Reinhold et al. [30], based on their better performance when used in DL models (see Section 4.1). Additional results obtained from other normalization techniques are available in Supplementary Materials (see Table S1 in Supplementary Materials).

### 3.3. Network Architectures

#### 3.3.1. Segmentation Approaches

**Figure 3:**
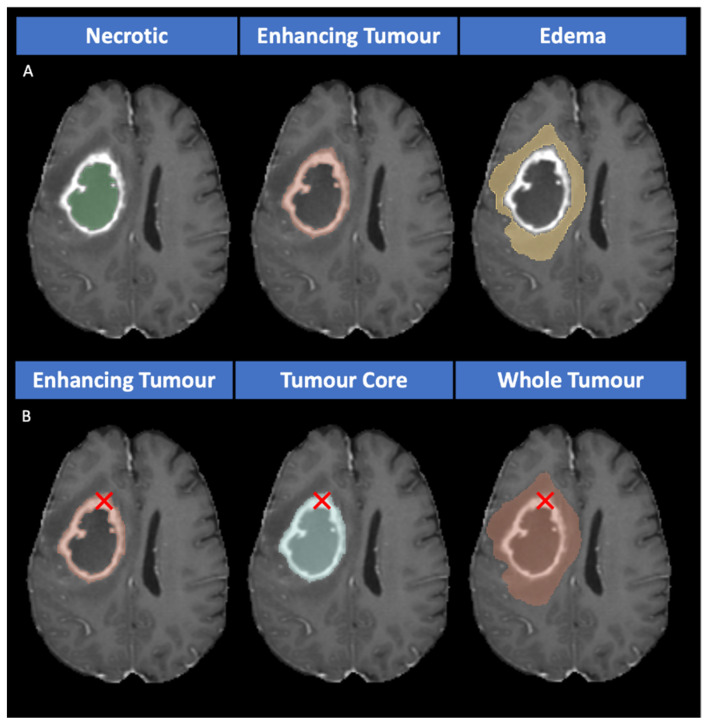
(**A**) The non-overlapping masks (the input for the multi-class approach) and (**B**) the overlapping masks (the input for the binary class and the multi-label approaches; the red cross shows an example of overlapping pixels for enhancing tumor tissue).

The segmentation strategy we propose employs three distinct approaches: multi-class segmentation, multi-label segmentation, and binary class segmentation. In the multi-class approach, non-overlapping masks were utilized, whereas for both the binary class and multi-label approaches, overlapping masks were employed, as illustrated in Figure 3.

**Figure 4:**
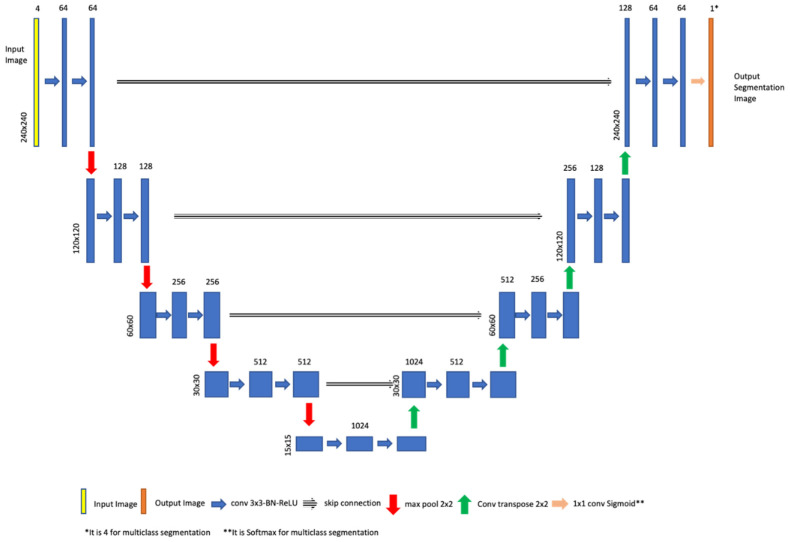
The proposed 2D UNET model.

We used three types of U-net architectures: 2D, 2.5D, and 3D U-net. The 2D U-net architecture is shown in Figure 4 whilst the 3D U-net architecture is the same architecture proposed by Cicek et al. [39].

**Figure 5:**
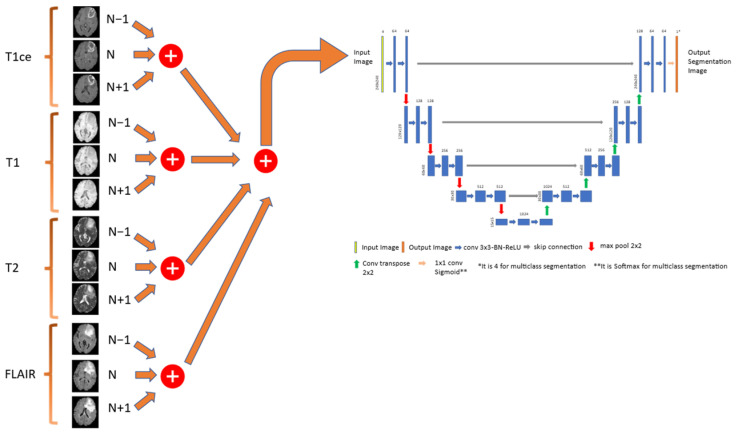
An example of each modality based on the 3-channel method of the 2.5D UNET model.

The 2.5D U-net architecture [38] employs the same 2D U-net model but enhances it to accommodate multi-channel inputs. Specifically, it utilizes a 3-channel method, where the current image, the previous image, and the following image in the volume are concatenated. This allows for the utilization of all MRI modalities as input. The application of the 3-channel method for each modality is depicted in Figure 5.

The proposed 2D U-net model has three versions. The only difference in the architecture lies in the last layer, where sigmoid is applied for binary class and multi-label segmentation, while softmax is utilized for multi-class segmentation. The activation function selection is related to the input mask.

The input image dimensions for the 2D model are 240 × 240 × 4, whereas for the 2.5D model, they are 240 × 240 × 12. These input images have a resolution of 240 × 240 pixels and include the concatenation of four registered modalities (T1, T1ce, T2, and FLAIR) for the third dimension. In the encoder path, there are four blocks, each utilizing convolutional layers and incorporating batch normalization for feature map normalization, followed by a ReLU activation layer. After the application of two convolutional layers, a max-pooling layer is employed. The initial feature maps start with 64 channels and are doubled after each max-pooling operation. Furthermore, the bottleneck kernel consists of 1024 channels. In the decoder path, a convolutional transpose layer is applied following two convolutional layers. Skip connections connect the encoder and decoder paths. Finally, the output layer produces a segmentation with a size of 240 × 240 pixels.

In the multi-class approach, as illustrated in Figure 2A, mutually exclusive classes (see Figure 3A) are considered. The architecture for this approach incorporates the softmax function in the last layer to handle non-overlapping masks. The masks for both the multi-label (see Figure 2B) and binary class (see Figure 2C) approaches, which are characterized by their non-mutually exclusive nature, are presented in Figure 3B. In the architectural design of these approaches, the sigmoid function is used in the last layer to accommodate overlapping masks. It is worth noting that the proposed 3D U-net model and nnU-net (DynU-net of utilizing MONAI [40]) also employ these three approaches, all with the same parameters, including the last layers and channels. The input patch size is set to 192 × 192 × 128 for each version. The patch size in the 3D models was increased from 128 × 128 × 128 to 192 × 192 × 128, shifting the focus towards capturing global information instead of local information and aiming for an optimized, time-efficient achievement of the optimum dice score. The increase in patch size allows our models to process larger data volumes per iteration, reducing the number of iterations needed for the entire dataset and streamlining the training process by enabling faster convergence. However, it is clear that this trade-off might result in poor generalizability and overfitting. The original patch size of nnU-net required more training time, which did not align with the research’s efficiency goals. This approach against time efficiency was also adopted by the extended nnU-net model, and we have provided a comparison of these methods in the Section 4.

#### 3.3.2. Loss Function

The loss function employed in all the previously mentioned U-net architectures is chosen as follows: the binary cross-entropy for multi-label/binary class segmentations and multi-class cross-entropy for multi-class segmentation. The selection of these two loss functions is based on the nature of the input masks, distinguishing between mutually exclusive and non-mutually exclusive classes (see Figure 3).

The choice of activation function is closely linked to the type of input mask used. For multi-class segmentation with non-overlapping input masks (as illustrated in Figure 3A), the softmax function is employed for each class. This approach ensures that the combined probabilities for all classes sum to 1, highlighting their interdependence. Consequently, if the probability of one class increases, it necessitates a corresponding decrease in the probabilities of the other classes. Based on this consideration we implemented a multi-class cross-entropy as loss function. Such function measures the similarity between the predicted probabilities and the true labels for each class interdependently. For a given pixel and N classes, the multi-class cross-entropy loss is;

(1) $$-\sum_{c=1}^{N}y_{o,c}\log\left(p_{o,c}\right)$$

where, $y_{o,c}$ represents a binary indicator (0 or 1) if class label c is the correct classification for pixel o and $p_{o,c}$ is the predicted probability that pixel o belongs to class c. For an entire image, the total loss is the average over all pixels. After the calculation of the loss function, the multi-class provides a detailed analysis with class imbalance issues, more suitable for complex tasks such as the provided input mask including ET, TC, and WT. 

In binary class segmentation, each input mask is processed individually using the sigmoid function. On the other hand, for multi-label due to utilizing overlapping input masks (c.f. Figure 3B), the sigmoid function is used. For the sigmoid function, each class prediction is independent of others; you obtain separate probabilities for each class. It can be applied to each label in a multi-label problem, treating each label as a separate binary classification. Then the average of each loss is used. For a single pixel, the binary cross-entropy loss is;

(2) −[ylog(p)+(1−y)log(1−p)](y)log(1−p)]

where, *y* represents the true label for the pixel (1 if it belongs to the object, 0 if it belongs to the background) and *p* is the predicted probability that the pixel belongs to the object. For an entire image, the total loss is the average of the losses for all pixels. This is for only the binary class segmentation approach. After the loss function calculation, the binary class provides computationally friendly simple analysis like the provided input mask having only one region for each training. For the multi-label segmentation approach, additionally, the calculated losses for each region are averaged to obtain a total loss. The multi-label segmentation approach provides complex and flexible analysis compared to others. However, this increases complexity, computational resources, and label correlation challenges.

Each segmentation approach has its inherent advantages and drawbacks. The conventional methods typically employ the same segmentation approach to delineate all tumor regions. Our solution diverges from this, by advocating for a region-focused ensemble learning model. This model assimilates the most efficacious segmentation approaches, each complemented by a distinct normalization technique, thereby leveraging the strengths of each to achieve superior performance on the segmentation of the targeted tumor region.

### 3.4. Dataset

In this paper, two different datasets are utilized for the purposes of training and testing the proposed approaches. The first one is the BraTS dataset which defines the retrospective collection of brain tumor MRI scans from multiple institutions [21,41,42]. In 2021, the BraTS contained 1251 training, 219 validation, and 570 testing data. The modalities in the BraTS are T1, T1ce, T2, and FLAIR. The delineations are performed manually by neuro-radiologists. 

In the BraTS competition, there are 3 different sub-tumors for GBM shown in Figure 2, namely, necrotic tumor (NCR, label 1), peritumoral edematous/invaded tissue (ED, label 2), and enhancing tumor (ET, label 4). There are 3 different sub-regions which overlap with each other according to the BraTS competition. These are ET (label 4), TC (label 1 + label 4), and WT (label 1+ label 2+ label 4). ET shows hyper-intensity in post-contrast T1-weighted (T1Gd) compared to T1. TC is considered for surgical excision and this region can be detected thanks to being hypo-intense in T1Gd [21]. WT shows abnormal hyper-intensity in the T2-flair sequence. For each patient’s MRI scans on the BraTS dataset, skull-stripping and co-registration for the same anatomical template are performed. Each modality has an isotropic resolution of 1 mm^3^ and a matrix size of 240 × 240 × 155.

The second dataset utilized in this study is the local dataset (STORM_GLIO), collected between April 2014 and April 2018 in Wales. This dataset comprises 108 glioblastoma patients, with only 53 of them having all four modalities similar to the BraTS dataset. Notably, the MRI scan format for STORM_GLIO is DICOM, differing from the format of the BraTS dataset. The resolution and matrix size of each patient is different. Additionally, these specifications can vary inter-modalities. For further details, please refer to Supplementary Materials.

### 3.5. Data Pre-Processing

**Figure 6:**
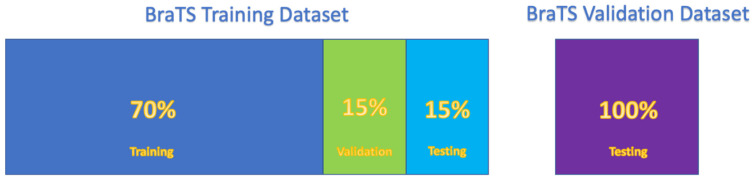
The use of the BraTS training and validation datasets.

The 3D MRI of the BraTS 2021 dataset has the dimension of 240 × 240 × 155. Before the collection of the slices (240 × 240) or patches (192 × 192 × 128), the training dataset samples are split into 70% training, 15% validation, and 15% testing shown in Figure 6. The training dataset is 10% less than the state-of-the-art model [29]. Following the train/test splitting, 155 slices are collected for each patient of the 2D U-net models. The slice collection is performed with a 3-channel method for 2.5D U-net. All slices are selected with the 3-channel method (the current (N), the previous (N − 1), and the following slices (N + 1)) for each modality. The first and the last slices of a patient (when there was no slice before or after the slice) are collected with the addition of a synthetic slice of fully black pixels. In 3D U-net, a patch of 192 × 192 × 128 is collected for each patient. 

Since STORM_GLIO is a clinical dataset, it included scans of different dimensions and a patient co-ordinate system that needs to be registered before use. Original MRI scans of the BraTS dataset are registered based on T1ce and SRI24 Atlas [43] (aligning different modalities in the same co-ordinate), skull stripped, and segmented to sub-tumor classes. To match our local dataset (STORM_GLIO) to the BraTS format, STORM_GLIO is applied to the BraTS pipeline with two adjustments. First, CaPTk [44,45] is used to make STORM_GLIO dataset match the BraTS dataset specifications. Instead of using CaPTk for skull stripping, HD-BET [46], which outperformed up-to-date publicly available brain extraction algorithms, is utilized. Second, the SRI-24 atlas registration is not applied to STORM_GLIO due to the deformation of ground truth. Because of the adjustments of the pre-processing steps, we managed to convert output segmentations from DL models to radiation therapy (RT) struct DICOM format or RTSTRUCT to be used in clinical applications. Additionally, MRI acquisition parameters of STORM_GLIO were added in Supplementary Materials.

### 3.6. The Details of the Implementation

For the 2D and 2.5D U-net models, the epoch number and batch size are selected as 100 and 16, respectively. Adam optimization [47] is performed with a learning rate of 0.0001. The augmentation process included rotating, horizontal flipping, and vertical flipping. The model is trained on an NVIDIA RTX 3070 GPU with 8 GB RAM, i7 11700 cpu, 32 GB RAM. For the 3D U-net and nnU-net models, the epoch number is 150, and the batch size is 4. Adam optimization is performed with a learning rate of 0.0001. The model is trained on an NVIDIA RTX 3090 GPU with 24 GB RAM, Intel i7 11700 cpu, 32 GB RAM. The Python v3.9.13, Pytorch framework (v1.10) on a Linux OS environment are utilized for the experiments. The Docker image for extended nnU-net [29], which secured first place in the BraTS 2021 challenge, has been obtained and is included for comparative purposes in this research. This Docker image encompasses 10 distinct models along with additional post-processing steps. All 3D models necessitate the resampling of our local datasets to achieve an isotropic resolution of 1 mm^3^ and a matrix size of 240 × 240 × 155 for optimal segmentation results. Following the collection of outputs, it is essential to resample the segmentations back to their original resolution and matrix size for the sake of comparison with the ground truth. 

To evaluate the results from the segmentation, the DSC score is utilized. The overlapping area between the ground truth and the prediction is compared with the total area of the ground truth and the prediction [48]. This calculation defines the DSC score. The score is between zero and one. A score of 1.0 describes the best-matched segmentation. $Y_{true}$ is for the ground truth and $Y_{pred}$ is for the prediction. The following equation shows the calculation of the DSC score. The DSC score is equal to one when neither the mask nor the predicted segmentations contain a pixel for tumor delineation [25]. 

All metrics are calculated by using a python library: Segmentationmetrics v1.0.1. For the targeted region, *DSC*, *sensitivity*, *specificity*, and 95% Hausdorff distance (*HD*95) are computed as follows:

(3) $$DSC=\frac{2|Y_{true,pos}\cap^{}\;Y_{pred,pos}|}{\left|Y_{true,pos}\right|+\left|Y_{pred,pos}\right|}$$

(4) $$Sensitivity=\frac{\left|Y_{true,pos}\cap^{}\;Y_{pred,pos}\right|}{\left|Y_{true,pos}\right|}$$

(5) $$Specificity=\frac{\left|Y_{true,neg}\cap^{}\;Y_{pred,neg}\right|}{\left|Y_{true,neg}\right|}$$

(6) $$HD95=95th\;percentile\;of\{\max_{a\in Y_{true,pos}}\min_{b\in Y_{pred,pos}\;}\left\|a\;-b{\|}_{2},\max_{b\in Y_{pred,pos}}\min_{a\in Y_{true,pos}\;}\right\|a-b{\|}_{2}\}\;$$

## 4. Results and Discussion

We evaluated the proposed method through three principal test scenarios. Initially, to identify the top-performing architecture among the models, the BraTS 2021 dataset was employed. A comparative analysis of each model was conducted to facilitate the model selection phase for the RFS+ strategy. Subsequently, the BraTS validation dataset was utilized to contrast the proposed U-net with the extended nnU-net (the winning model of BraTS 2021). Ultimately, in the third experimental scenario, we executed the RFS+ strategy with the highest-performing architectures against both the extended nnU-net and the models devoid of the RFS+ strategy.

### 4.1. Model Selection via the BraTS 2021 Dataset

This section presents experiments, evaluations, and comparisons of the proposed DL models with different approaches using several intensity normalization techniques. 

The effectiveness of the proposed models was tested with the BraTS 2021 and STORM_GLIO datasets and compared to each other in order to pursue a model selection step. The results of the aforementioned analysis are presented in Table 1.

On STORM_GLIO, Table 2 shows the segmentation results of the models trained on the BraTS dataset with a multi-class segmentation approach via Z-score normalization.

According to Table 1 and Table 2, the highest DSC scores for TC and GTV are both obtained by the 2D U-net model. The 2D model uses only one slice which might result in higher DSC scores. The additional slices for 2.5D and 3D might decrease the segmentation accuracy due to different slice thicknesses and resolutions. Table 3 compares the models of 2D U-net with different approaches and normalization techniques quantitively with the DSC score for the BraTS 2021 training dataset. 

Investigating the results presented in Table 3 with the focused region of TC, we concluded that the three high-performing models based on DSC score are Nyul-binary class (89.42%), Z-score-multi-class (89.71%) and Z-score-binary class (89.48%). In the proposed method, the three models are then fed to a weighted average ensemble learning stage.

### 4.2. Benchmarking RFS+ Method: Comparative Study to the BraTS 2021 Winner Model

On the BraTS 2021 validation dataset, the evaluation in this section will compare the performance of the extended nnU-net and the proposed U-net variants and nnU-net models. Intensity normalization is the Z-score for the proposed U-net variants and nnU-net model. Table 4 shows the comparison of DL models for the BraTS validation dataset.

As can be seen in Table 4, the extended nnU-net [29] outperformed the proposed models for each region. This model has been developed on the U-net model with modifications (nnU-net) based on the BraTS training dataset which has a fixed size and resolution. The matrix size and resolution of the BraTS validation dataset are fixed, as well. Thus, the extended nnU-net easily achieved better DSC scores over the proposed U-net variants and the nnU-net model for each sub-region. The ground truth for the BraTS validation dataset is not shared publicly. To obtain DSC scores, all evaluations need to be tested online. The comparisons are shown in DSC scores with segmentation predictions for U-NET variants, the nnU-net, and the extended nnU-net model. 

### 4.3. Ablation Study

To improve the performance of the ensemble learning, several combinations of segmentation approaches and normalization techniques were employed. To demonstrate the importance of each segmentation approach, an ablation study was conducted. The U-net model employing the multi-class segmentation approach served as the baseline (base U-net) due to the nature of the training dataset masks (non-overlapping).

**Figure 7:**
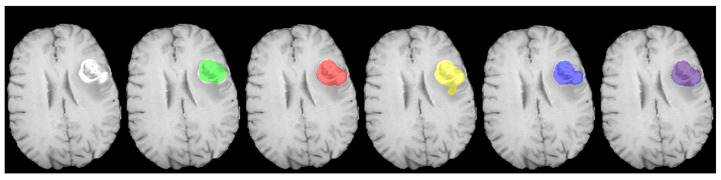
The predictions of models on STORM_GLIO. From left to right: T1ce, ground truth, 2D U-net Nyul/binary class, 2D U-net Z-score/multi-class, 2D U-net Z-score/binary, and 2D U-net with RFS+.

Table 5 presents the performance of different U-net models, incorporating varying segmentation approaches and normalization techniques as part of our proposed RFS+ strategy on the STORM_GLIO dataset. The base U-net rows in Table 5 provide a benchmark for performance comparison using the Dice score. Each column corresponds to a unique combination of segmentation approach and normalization technique, with the final column displaying Dice scores for GTV segmentation. The table reveals that base U-net achieves the highest Dice score among U-net models using Z-score normalization. Switching to Nyul normalization, the binary class model outperforms others. Notably, while no single model consistently delivers the highest Dice score, each combination of the segmentation approach and normalization technique uniquely addresses different aspects of the target area (see Figure 7). The RFS method, employing a union approach that combines segmentations from all models into one contour, showed a modest increase in the Dice score from 78.43% to 78.51% [38]. The RFS+ further incorporates a weighted ensemble learning method, with weights calculated based on the BraTS training dataset results. This approach, particularly when applying the models using Z-score normalization in ensemble learning, led to a slight improvement in Dice score to 78.69%. Upon integrating Nyul normalization within RFS+, we selected the top three models based on their Dice scores from the BraTS training data. The proposed RFS+ strategy boosted the Dice score on STORM_GLIO from 78.43% to 79.22%, marking the highest achievement in GTV segmentation. This improvement underscores the effectiveness of diversifying the base U-net model with various normalization and segmentation combinations, significantly enhancing performance through ensemble learning.

### 4.4. Validating RFS+ on Local Dataset

This section presents the usage of the proposed strategy—RFS+—for the purposes of GTV segmentation for the local dataset. We tested the best-performing U-net and nnU-net models with and without RFS+ along with the extended nnU-net model. Additionally, the predecessor method (the RFS) [12] for 2D U-net is compared. DSC scores, HD95, sensitivity and specificity for GTV segmentation of each model are given in Table 6.

Upon scrutinizing the outcomes detailed in Table 6, it can be concluded that the 2D U-net model, integrated with RFS+, surpassed the extended nnU-net model. This is achieved by adopting the combination of the top three high-performing models identified in the preceding section. Notably, the 2D U-net with RFS+ covers all three approaches of multi-class, multi-label, and binary class along with incorporating both Nyul and Z-score normalization techniques. In contrast, the extended nnU-net model only utilizes the multi-label approach and Z-score normalization technique. Therefore, our result with the ensemble learning within RFS+ achieves a higher DSC score on GTV segmentation and provides a considerable generalization capability compared to the reference models. Furthermore, the implementation of RFS+ has led to an increase in DSC of up to 1% compared to the RFS. Due to the demonstration of accurate delineation of the tumor margins, lower HD95 of segmentation is important in the planning of both surgical and radiotherapy treatments. The comparative analysis between the proposed RFS+ model and the current state-of-the-art model in terms of HD95 reveals a small difference (8.1 vs. 7.8). This indicates that the RFS+ can yield adequate results for clinical applications while taking efficiency into consideration. In clinical settings, when including computer-aided decision-making processes for diagnoses and evaluation purposes, a higher sensitivity might be required for comprehensive coverage and assessment of all tumor tissues. The RFS+ exhibits a notable enhancement in sensitivity compared to the state-of-the-art model, elevating it from 74.07 to 76.93. Accurately distinguishing healthy tissue from tumor tissue is vital, especially during radiotherapy and surgical procedures. Based on this, high sensitivity and specificity is important during treatment planning and response assessments to correctly identify tumor tissue and differentiate this from healthy brain tissue. The RFS+ model exhibits considerable promise in safeguarding healthy tissue, emphasizing efficiency in both time and memory consumption when compared to the leading model (with specificity 99.97 vs. 99.95). 

Figure 7 demonstrates the prediction of 2D U-net models with and without RFS+. Each model without RFS+ segmented the tumorous tissue differently. No single approach exhibited unequivocal superiority over the others; rather, each approach demonstrated unique advantages depending on the specific task requirements, such as TC/GTV segmentation. RFS+ yields improved segmentation by encompassing the benefits of each individual approach comprehensively.

**Figure 8:**
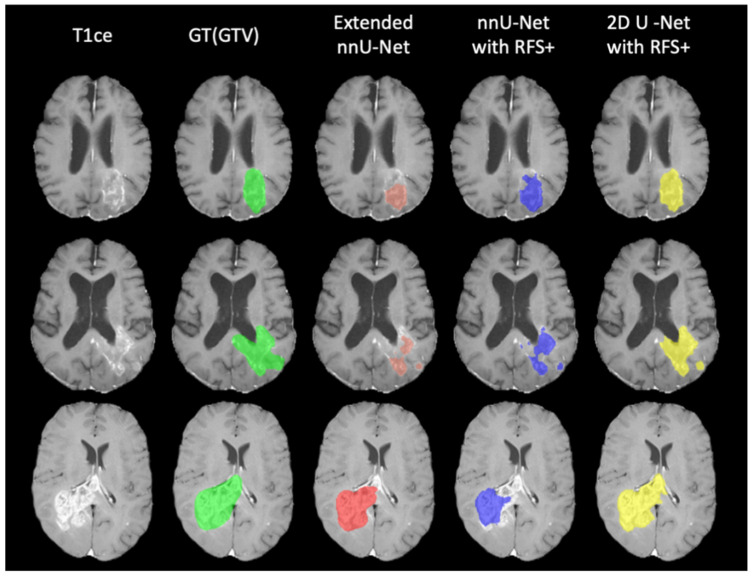
The predictions of models on STORM_GLIO. From left to right: T1ce, ground truth (GTV), extended nnU-net model, nnU-net with RFS+, and 2D U-net with RFS+.

The comparative illustration between the 2D U-net model with RFS+ and the extended nnU-net model is depicted in Figure 8. It is evident from the figure that the proposed model accurately segmented a larger amount of tumor tissue compared to the winner model. The resolution and matrix size of the local dataset differ for each patient and can even vary between modalities for the same patient, presenting additional challenges to the segmentation task. Therefore, the model incorporating RFS+ demonstrates superior generalizability over the winning model on the local dataset, proving its resilience and adaptability to diverse and complex datasets.

The RFS+ strategy marks a significant innovation in brain tumor segmentation, notably augmenting the adaptability of DL models within clinical environments. Compared to the current state-of-the-art model, the RFS+ on 2D U-net models exhibits remarkable efficiency, necessitating 10% less training dataset, 67% less memory, and reducing training time by 92% (see Tables S2–S4 in Supplementary Materials), thereby presenting a substantial advancement in computational resource management. In the analysis of a local dataset, the RFS+ achieved a Dice score of 79.22%, a metric that stands out in the context of brain tumor segmentation tasks and underscores the model’s accuracy. The RFS+ incorporates an ensemble learning approach, combining various models and employing diverse normalization techniques, which collectively contribute to the enhancement of segmentation accuracy.

The RFS+ appears to address dataset variability and generalization more explicitly than DeepMedic (v0.8.4), which is crucial for real-world clinical applicability. The multi-scale approach of DeepMedic (v0.8.4), which captures both local and global contextual details, presents a potential area of limitation for the RFS+. The refined, cascading structure of cascade U-net is tailored for precise tumor boundary delineation, which could potentially yield more accurate segmentations compared to those by the RFS+. Cascade U-net’s layered methodology may exhibit superior performance in managing intricate tumor structures. 3D-DSN employs a deep supervision mechanism, ensuring comprehensive feature extraction at multiple levels, potentially resulting in more detailed segmentations than those achieved by the RFS+ approach. The capacity of 3D-DSN to extract learning from its intermediate layers might endow it with an edge in identifying subtle features, a facet that could pose a challenge for RFS+, depending on its learning methodology. The nnU-net’s proven ability to generalize across various medical segmentation tasks stands in contrast to RFS+, which has yet to demonstrate similar breadth in generalizability across diverse datasets and segmentation challenges. Known for its automated configuration, nnU-net can adjust to different tasks and datasets: this is a level of adaptability and automation that RFS+ has not yet developed. The nnU-net has undergone rigorous validation and benchmarking on multiple datasets, including the well-regarded medical segmentation decathlon, whereas RFS+ is yet to be as comprehensively validated, particularly on diverse and complex datasets. Demonstrating robustness under a variety of imaging conditions and with different medical image types, nnU-net’s versatility might present a benchmark for RFS+, whose robustness in such a wide array of conditions necessitates further investigation. With a design conducive to scaling effectively across different tasks and dataset sizes, nnU-net’s flexibility might highlight potential scaling or adaptability challenges for RFS+ in handling exceptionally large or complex datasets. Although the RFS+ strategy demonstrates promising results when compared with the leading model, the primary benchmark used for comparison is the nnU-net. Future work should involve more comprehensive analyses involving a wider array of state-of-the-art models. The performance on standard datasets like BraTS is well established, but its effectiveness on a wider range of datasets might need more exploration. The varying clinical environments present a challenge for the generalizability of DL models, including those using RFS+. Our study focused on the GTV/TC label which include both ET and NCR labels from our local dataset. Finally, the effect of intensity normalization on outcomes differs, based on the specific local dataset and this is an area that necessitates additional research.

## 5. Conclusions

This study highlights the benefits of adopting the RFS+ strategy for the purposes of brain tumor segmentation in MR images. Although the extended nnU-net resulted in the best DSC score on the BraTS validation dataset, the performance of the proposed 2D U-net model with RFS+ strategy on GTV delineation had the best DSC score in the local dataset. The main reason for this is that the local dataset has no fixed resolution and matrix size for each patient, unlike the BraTS dataset. It is necessary to determine which normalization technique will be most effective for the targeted sub-tumor which is TC/GTV in this research. When using U-net with a multi-class approach on the BraTS 2021 validation dataset, the best DSC scores among the proposed models are 77.45%, 82.87%, and 90.82% for ET, TC, and WT segmentation, respectively. The model incorporating RFS+ yields the best DSC score on our local dataset, achieving 79.22% for GTV segmentation. Moreover, it is worth noting that the model requires 10% less training dataset, 67% less memory, and takes 92% less time for training, as detailed in Supplementary Materials (see Tables S2–S4 in Supplementary Materials).

In conclusion, this study effectively improves segmentation accuracy on the local dataset with our proposed strategy RFS+, setting the stage for future state-of-the-art models to similar benefits from the RFS+ strategy for enhanced generalizability in brain tumor segmentation. The RFS+ model shows encouraging progress, especially in its computational efficiency and ability to adapt to varied clinical contexts. However, to comprehensively assess its place in the domain of brain tumor segmentation, it is essential to conduct further validations and engage in more extensive comparisons with a diverse set of algorithms, thereby mitigating the risk of potential biases. We are planning to extend the application of RFS+ to not only time and memory efficiency but also performance improvement in brain tumor segmentation compared to a wider array of state-of-the-art models, under different clinical settings. The development of data augmentation methods for clinical applications is crucial to align training data with real-world data.

## Figures and Tables

**Table 1:** The comparison of DSC Scores for the 2D, 2.5D, 3D U-NET, and nnU-net with Z-score normalization and multi-class approach on the BraTS 2021 training dataset.

Model	ET	TC	WT
nnU-net	83.96	88.34	92.53
3D U-net	83.21	87.55	91.67
2.5D U-net	84.34	88.55	91.64
2D U-net	84.99	89.71	91.65

**Table 2:** The comparison of the models with multi-class approach on STORM_GLIO.

Models	GTV
nnU-net	77.45
3D U-net	75.74
2.5D U-net	70.35
2D U-net	78.43

**Table 3:** The comparison of DSC scores for binary class, multi-label, and multi-class approaches of 2D U-net with several intensity normalization techniques on the BraTS 2021 training dataset.

Intensity Norm. Tech	Segmentation Approach	ET	TC	WT
Nyul	multi-class	79.44	79.53	88.98
	multi-label	83.52	88.78	92.05
	binary class	84.21	89.42	90.30
Z-score	multi-class	84.99	89.71	91.65
	multi-label	82.29	87.27	92.24
	binary class	85.19	89.48	92.18

**Table 4:** The comparison of the BraTS validation dataset based on online evaluation.

Models	DSC(ET) (%)	DSC(TC) (%)	DSC(WT) (%)
Extended nnU-net [27]	84.51	87.81	92.75
nnU-net	78.65	85.96	91.67
3D U-net	78.89	81.05	91.16
2.5D U-net	78.80	84.23	90.90
2D U-net	77.45	82.14	90.82

**Table 5:** The ablation study on U-net.

	Z-Score Normalization			Nyul Normalization			Combined Method		GTV Dice Score (%)
	Multi-Class	Multi-Label	Binary	Multi-Class	Multi-Label	Binary	Union	Ensemble	
Base U-net (Multi-class)	Yes								78.43
Multi-label		Yes							77.91
Binary			Yes						78.22
Base U-net (Multi-class)				Yes					77.61
Multi-label					Yes				78.20
Binary						Yes			78.91
RFS	Yes	Yes	Yes				Yes		78.51
RFS+ (only Z-score normaliz-ation)	Yes	Yes	Yes					Yes	78.69
Proposed RFS+	Yes		Yes			Yes		Yes	79.22

**Table 6:** The comparison of base models, the proposed models, and the state-of-the-art model on the GTV label.

Models	DSC ↑	HD95 ↓	Sensitivity ↑	Specificity ↑
extended nnU-net [27]	79.09	7.8	74.07	99.97
nnU-net (base nnU-net)	77.83	10.72	74.65	99.95
nnU-net with RFS+	78.30	8.2	73.59	99.97
2D U-net (base U-net)	78.43	8.8	77.24	99.94
2D U-net with RFS [12]	78.51	11.33	78.48	99.93
2D U-net with RFS+	79.22	8.1	76.93	99.95

## Data Availability

The BraTS data used in this study are openly available and can be accessed from the following source: [21].

## Supplementary Materials

The following supporting information can be downloaded at: https://www.mdpi.com/article/10.3390/cancers15235620/s1, Figure S1: RFS+ for ET based on Table 1; Figure S2: RFS+ for TC based on Table 1; Figure S3: RFS+ for WT based on Table 1; Figure S4: (A) Multi-class segmentation (B)Multi-label segmentation (C) Binary class segmentation (D) RFS+ for ET.; Figure S5: (A) Multi-class segmentation (B) Multi-label segmentation (C) Binary class segmentation (D) RFS+ for TC; Figure S6: (A) Multi-class segmentation (B)Multi-label segmentation (C) Binary class segmentation (D) RFS+ for WT; Table S1: The results of RFS+ for ET, TC and WT; Table S2: The extended nnU-Net requirements; Table S3: The 2D U-Net with RFS+ requirements (Any region); Table S4: The comparison of the ensemble methods. References [29,30] are cited in the supplementary materials.
