# Supplementary material for: RFS+: A Clinically Adaptable and Computationally Efficient Strategy for Enhanced Brain Tumor Segmentation
Source: Cancers (Basel). 2023 Nov 28;15(23):5620. doi: 10.3390/cancers15235620 (PMC10705309; doi:10.3390/cancers15235620)
Supplement: Supplementary file 1 [file cancers-15-05620-s001.zip › cancers-2698895-supplementary.pdf]

Supplementary Materials: RFS+: A Clinically Adaptable and Computationally Efficient Strategy for Enhanced Brain Tumor Segmentation

Abdulkerim Duman, Oktay Karakuş, Xianfang Sun, Solly Thomas, James Powell and Emiliano Spezi

RFS+ for each region

A Normalization Techniques

For the BraTS dataset, several normalization techniques [1] are implemented in this study, categorized into two main types: individual time-point normalization methods and sample-based normalization methods. The individual time-point normalization methods incorporate Z-score normalization (zscore-normalize), Fuzzy C-means (FCM)-based tissue-based mean normalization (fcm-normalize), Kernel Density Estimate (KDE) WM mode normalization (kde-normalize), and WhiteStripe (ws-normalize). Meanwhile, the sample-based normalization methods include Least squares (LSQ) tissue mean normalization (lsq-normalize), Piecewise Linear Histogram Matching (nyul-normalize) except RAVEL (ravel-normalize) due to not applicable.

Table S1 shows the results of RFS+ on ET, TC, WT for each segmentation approach with variable normalization techniques.

Table S1. The results of RFS+ for ET, TC and WT.

Intensity norm. tech	Segmentation Approach	ET	TC	WT
Nyul	Multiclass	79.44	79.53	88.98
	Multi-label	83.52	88.78	92.05
	Binary class	84.21	89.42	90.30
Z-score	Multiclass	84.99	89.71	91.65
	Multi-label	82.29	87.27	92.24
	Binary class	85.19	89.48	92.18
Whitestripe	Multiclass	83.61	87.99	90.47
	Multi-label	83.05	88.17	91.77
	Binary class	84.12	88.24	91.83
FCM	Multiclass	78.65	78.23	88.67
	Multi-label	77.56	79.42	87.65
	Binary class	83.65	84.21	88.53
LSQ	Multiclass	78.59	78.04	87.32
	Multi-label	79.34	80.11	86.59
	Binary class	82.34	84.87	83.98
KDE	Multiclass	79.22	77.45	88.67
	Multi-label	81.03	78.66	87.45
	Binary class	84.17	88.22	88.34

B. RFS+ Workflows for each region.

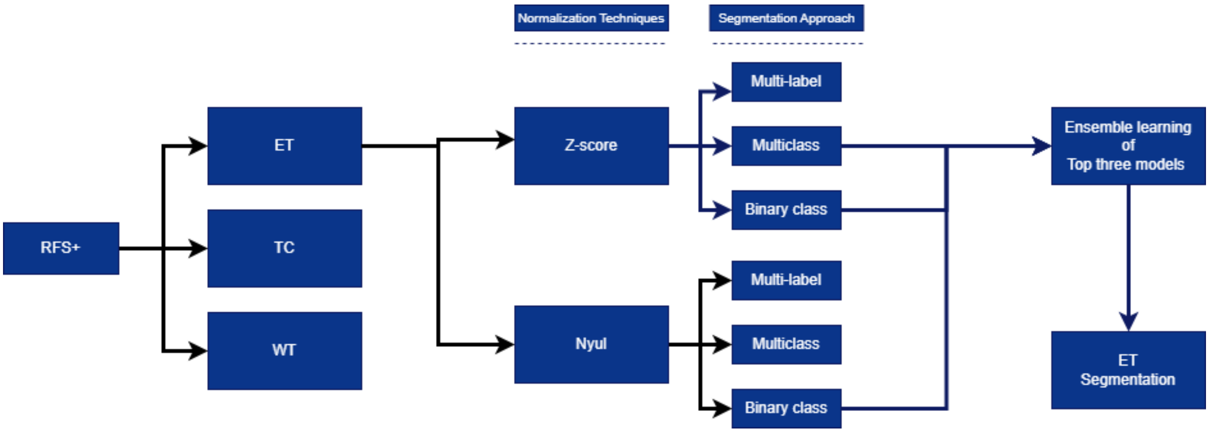

Figure S1. RFS+ for ET based on Table S1.

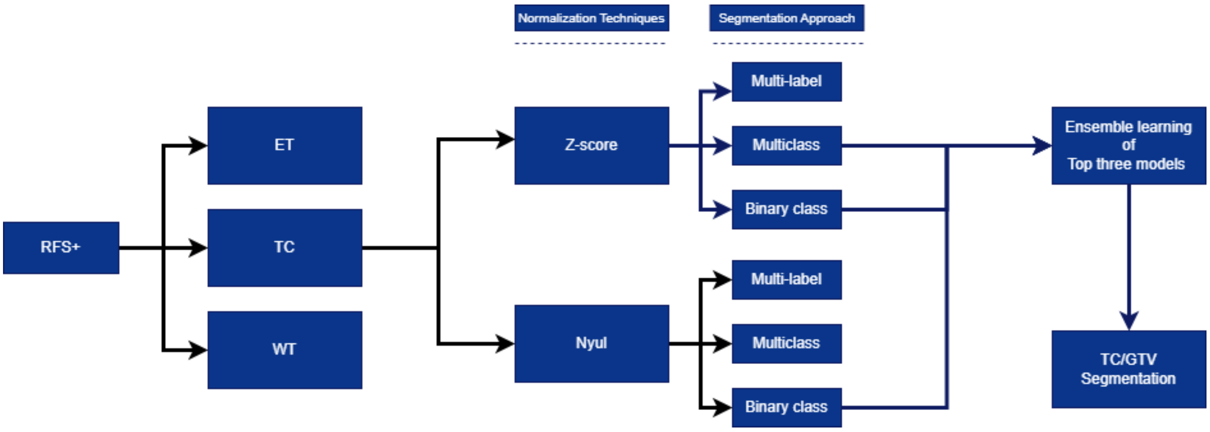

Figure S2. RFS+ for TC based on Table S1.

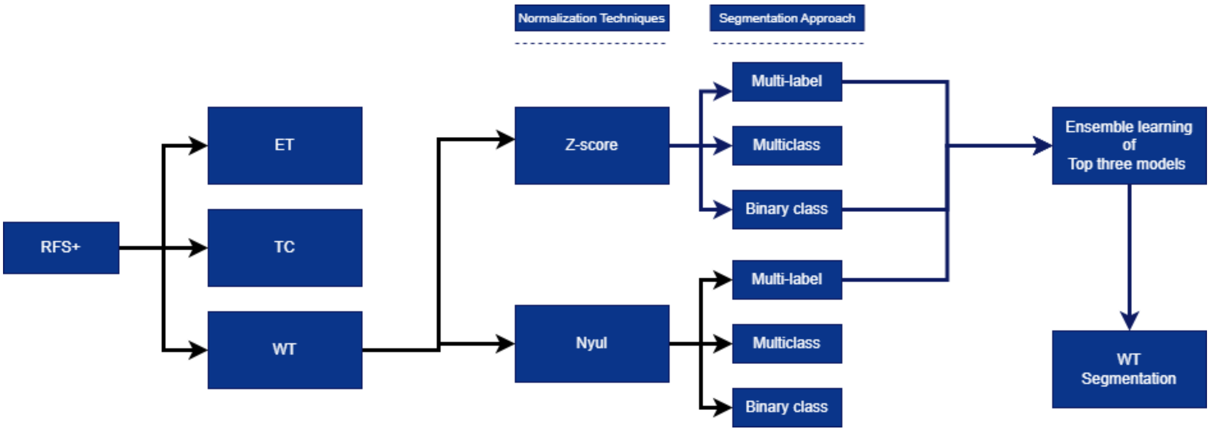

Figure S3. RFS+ for WT based on Table S1.

C RFS+ with each segmentation approach for each region

Figure S4 shows each segmentation approaches with their respective inputs and RFS+ for ET.

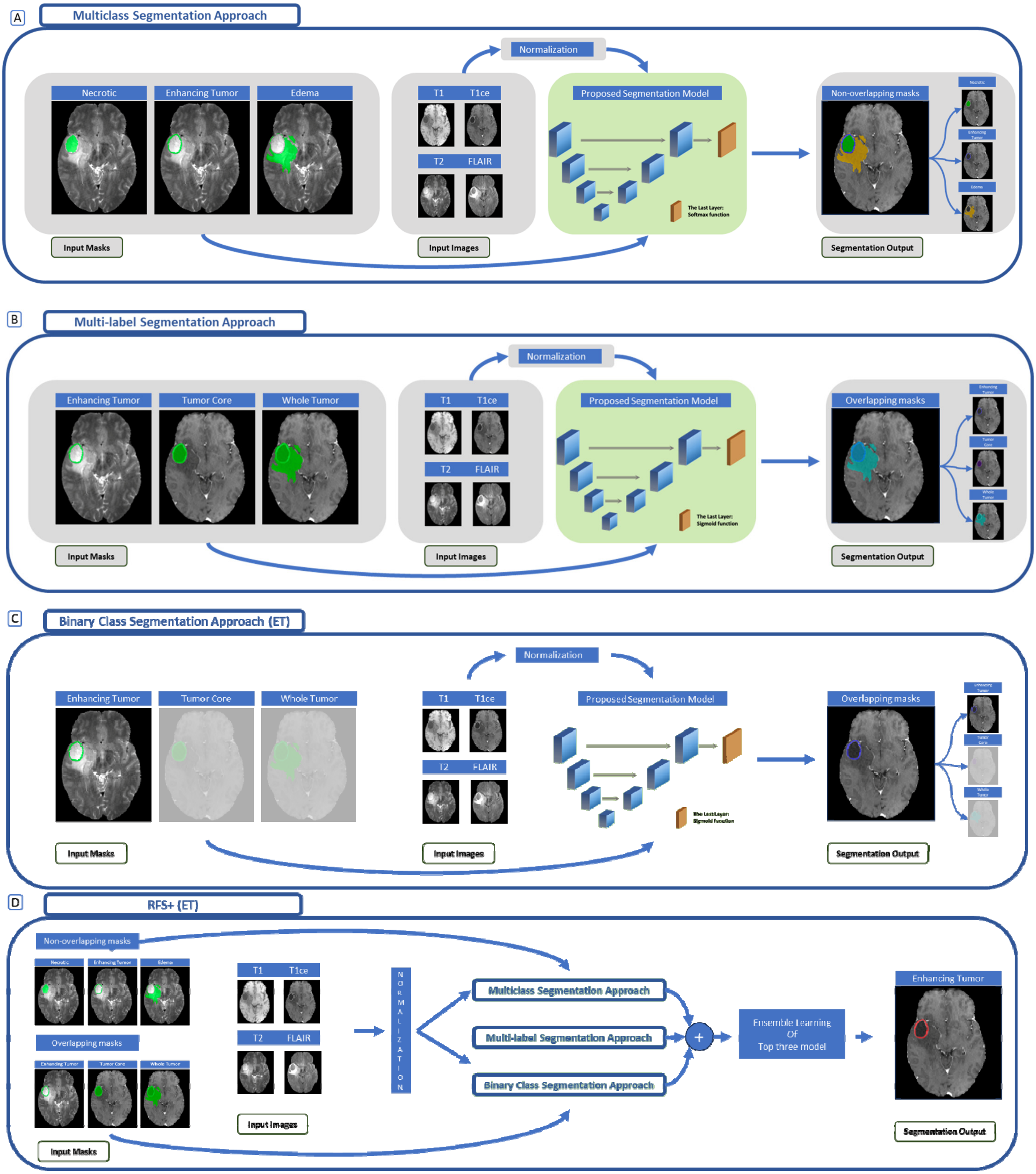

Figure S4. (A) Multiclass segmentation (B) Multi-label segmentation (C) Binary class segmentation (D) RFS+ for ET.

Figure S5 shows each segmentation approaches with their respective inputs and RFS+ for TC.

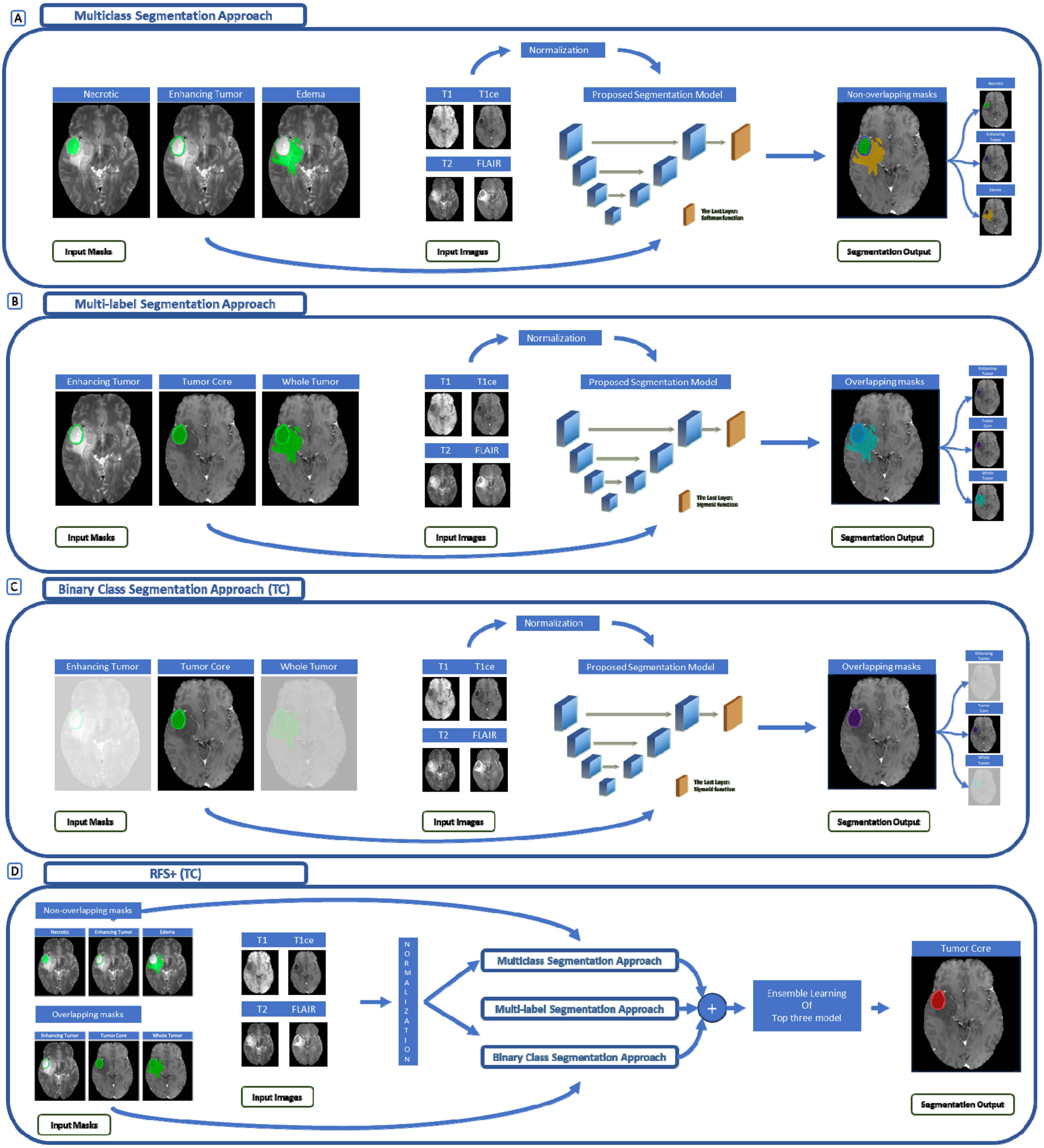

Figure S5. (A) Multiclass segmentation (B) Multi-label segmentation (C) Binary class segmentation (D) RFS+ for TC.

Figure S6 shows each segmentation approaches with their respective inputs and RFS+ for WT.

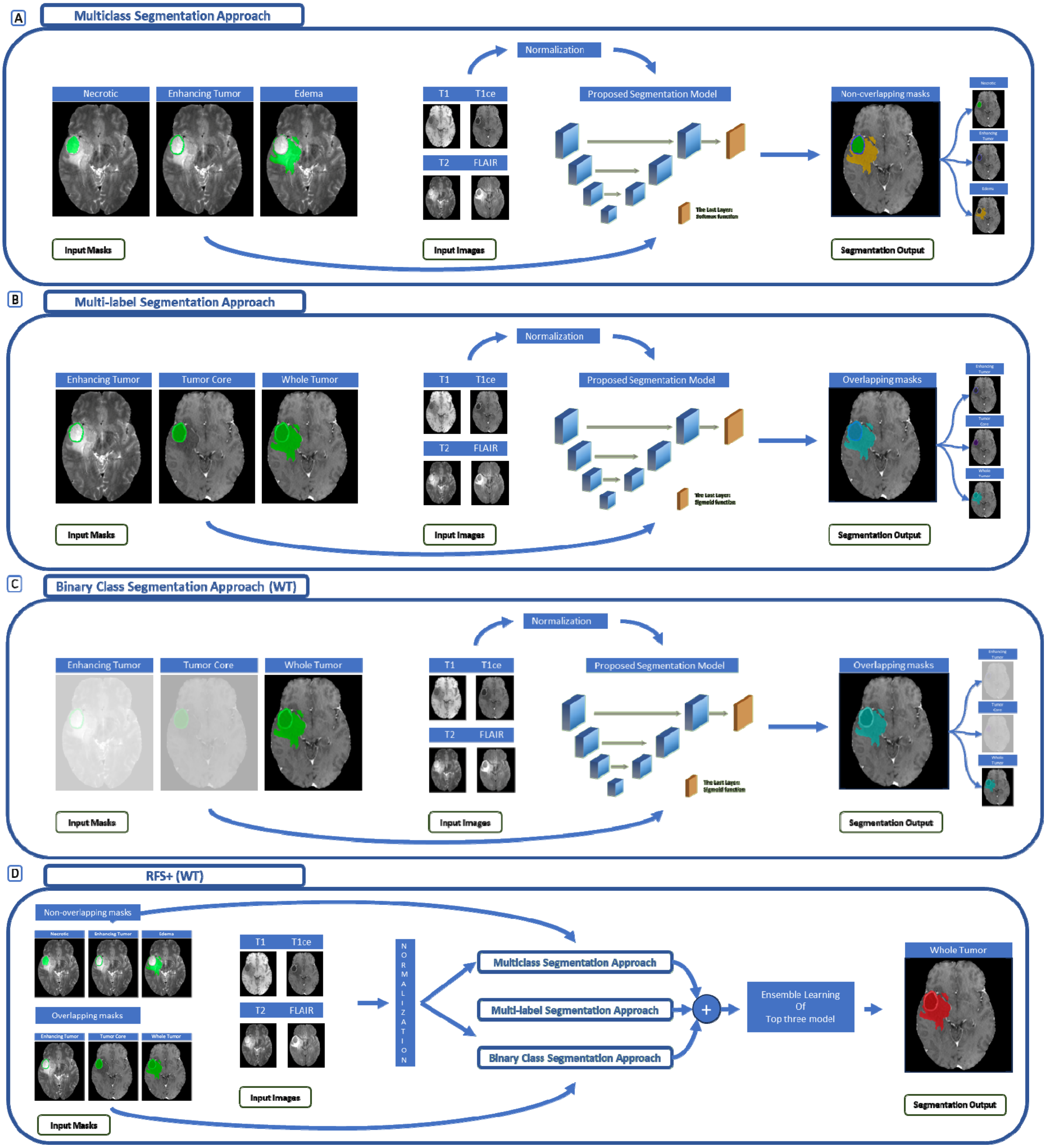

Figure S6. (A) Multiclass segmentation (B) Multi-label segmentation (C) Binary class segmentation (D) RFS+ for WT.

D Analyzing Training Requirements and Time Efficiency

Tables S2–S4 reveal that utilizing just 8GB of GPU memory and three days on an RTX 3090 is adequate to surpass the performance of the extended nnU-Net [2]. Notably,

the 2D U-Net model incorporating RFS+ demonstrates remarkable efficiency by requiring only 66% of the memory and completing training in 92% less time.

Table S2. The extended nnU-Net requirements.

	Models	RTX 3070 8GB		RTX 3090 24 GB		The model number	Total Time (Days)
		Trainable	Time in Days	Trainable	Time in Days		
Ensemble	BL baseline nnUNet	-	-	X	5	5	25
	BL+L+GN nnUNet with larger Unet	-	-	X	2	5	10
The extended nnU-Net			-				35

Table S3. The 2D U-Net with RFS+ requirements (Any region).

	Models	RTX 3070 8GB		The model number	Total Time (Days)	RTX 3090 24 GB		The model number	Total Time (Days)
		Trainable	Time in Days			Trainable	Time in Days		
Ensemble	2D U-Net multiclass (Z-score normalization)	X	3	1	3	X	1	1	1
	2D U-Net binary class (Z-score normalization)	X	3	1	3	X	1	1	1
	2D U-Net binary class (Nyul normalization)	X	3	1	3	X	1	1	1
RFS+					9				3

Table S4. The comparison of the ensemble methods.

Ensemble	RTX 3070 8GB		RTX 3090 24 GB	
	Trainable	Time in Days	Trainable	Time in Days
The extended nnU-Net	-	-	X	35
RFS+	x	9	x	3

E Acquisiton Parametres retrieved from DICOM for STORM_GLIO

	T1	T1ce	T2	FLAIR
Thickness/mm	4.77 +-0.47	4.76 +-0.47	4.74 +-0.56	4.81 +-0.39
Repetition time/ms	489 +-96	494 +-98	5627 +-1856	8084 +1832
Echo Time/ms	11 +-2	11 +-2	97 +-8	112 +-27
Inversion Time/ms	0 +-0	0 +-0	0 +-0	2217 +-259
Field Strength/T	1.54 +0.24	1.5 +-0	1.54 +0.24	1.54 +0.24
Rows	426 +-145	424 +-146	546 +-185	475 +-219
Columns	417 +-147	415 +-148	527 +-198	458 +-232
Pixel spacing/mm	0.62 +-0.19	0.62 +-0.19	0.48 +-0.14	0.59 +-0.21
Slice Spacing/mm	5.99 +-0.73	5.98 +-0.74	6.27 +-0.96	6.34 +-0.72
SAR	1.09 +-0.77	1.07+- 0.76	0.91 +-0.53	0.69 +-0.67

References

1. J.C. Reinhold, B.E. Dewey, A. Carass, J.L. Prince, Evaluating the impact of intensity normalization on MR image synthesis, in: Medical Imaging 2019: Image Processing, SPIE, 2019: pp. 890–898.
2. H.M. Luu, S.-H. Park, Extending nn-UNet for Brain Tumor Segmentation, in: A. Crimi, S. Bakas (Eds.), Brainlesion: Glioma, Multiple Sclerosis, Stroke and Traumatic Brain Injuries, Springer International Publishing, Cham, 2022: pp. 173–186.
